# Supplementary material for: Influence of physical activity on serum vitamin D levels in people with multiple sclerosis
Source: PLoS One. 2020 Jun 11;15(6):e0234333. doi: 10.1371/journal.pone.0234333 (PMC7289406; doi:10.1371/journal.pone.0234333)
Supplement: Attachment — Submitted filename: Response to Revision 1.docx [file pone.0234333.s003.docx]

Response to reviewer:

The authors submitted a revision of their manuscript, in which some minor textual changes were made, a figure was added, and the table was updated. The addition of the scatterplots is very helpful, the addition of clinical data is also very helpful to disclose that their cohort is not only very small but also very heterogeneous in many aspects.

Response: Thanks for carefully evaluating the revised manuscript.

1. The issue of not setting a significance level has not been solved. The authors correctly refer to other publications raising the discussion on the use of P-values, and reveal the instructions of a popular statistical program apparently provide some guidance. The severe lack of power of their dataset still receives a very limited role in the discussion of the validity of their results. The authors should at least be very careful in their formulation of study design and of conclusions they can and cannot draw from their data. This is most critical in their abstract:

*”The aim […] was to correlate” is less accurate than “The aim […] to explore correlations between”

*”In conclusion, physical activity has an independent effect on vitamin D levels” is less accurate than “in conclusion, physical activity has an effect on vitamin D levels independent of sunlight exposure time” since many other confounders were not assessed.

Response: We agree that the wording should be more careful and adopted the suggestions accordingly.

2. In their explanation of a lack of correlation between 25(OH)D and sun exposure time in the manuscript, the authors completely ignore that they have neglected a lot of potential very relevant confounders (see my first comments) and their proxy estimate of UVB light exposure may be very inaccurate. Explaining the lack of correlation only as ceiling effect is not valid. The authors should include a statement on this both at line 146 (were hey attribute the lack of any correlation to ceiling effect) and in the discussion section.

Response: We acknowledge the issue of confounders and have now added statements in the results and discussion sections. It is not that we ignored that fact, but it is hard to estimate how much of an effect those factors had.

3. I still have problems with the revised last line of their discussion: “Therefore, there is a dual effect of physical exercise on both, vitamin D serum levels and clinical improvement, whereas vitamin D supplementation can increase 25(OH)D3 serum levels but failed to show a clinical effect in a series of interventional trials.” This statement is still not supported by their data, further downtuning would be appropriate.

First, the authors did not show there is an effect of exercise on 25(OH)D levels in their cohort, but rather that there is a weak (not talking about significance) correlation between physical activity and 25(OH)D levels in their small cohort.

Response: True, we did not show an effect in terms of an interventional trial. The term effect is frequently used in context with effect size, strength of correlation or association, etc. However, we have changed the wording to correlation rather than effect.

Second, the authors refer to a meta-analysis from 2018 on vitamin D supplementation studies, but do ignore the two largest and most recently published trials on vitamin D supplementation in MS (Camu et al., N3 2018; Hupperts et al., Neurology 2018). The authors are correct on the observation that these studies did not make their primary endpoints. However, in secondary endpoints, and subgroup analysis, potential relevant signals were noted in relapse rate and MRI outcomes. The notion on MRI endpoints is further in line with an earlier small study by Soilu-Hanninen et al., JNNP 2012.

Response: We have added the 2 recently published studies by Camu et al and Hupperts et al. and changed the wording to “no effect on primary clinical outcomes”. As the reviewer rightly observes, the primary endpoints were not met in any of the interventional trials so far. We feel that evidence from primary outcomes outweigh secondary non-clinical outcomes and post-hoc subgroup analyses. A 5% significance level, which is most commonly used, by definition results in one out of 20 false positive results. The more post-hoc analyses one performs the higher the likelihood of such a false positive outcome. Altogether, 14 interventional trials (12 summarized in the Cochrane review, reference 29, and the 2 recent trials mentioned above) failed to meet the primary outcomes.
